# Mitochondrial Complex I activity signals antioxidant response through ERK5

**DOI:** 10.1038/s41598-018-23884-4

**Published:** 2018-05-09

**Authors:** Abrar Ul Haq Khan, Nerea Allende-Vega, Delphine Gitenay, Johan Garaude, Dang-Nghiem Vo, Sana Belkhala, Sabine Gerbal-Chaloin, Claire Gondeau, Martine Daujat-Chavanieu, Cécile Delettre, Stefania Orecchioni, Giovanna Talarico, Francesco Bertolini, Alberto Anel, José M. Cuezva, Jose A. Enriquez, Guillaume Cartron, Charles-Henri Lecellier, Javier Hernandez, Martin Villalba

**Affiliations:** 1 IRMB, INSERM, Univ Montpellier, Montpellier, France; 2 Institut de Regenerative Medicine et Biothérapie (IRMB), CHU Montpellier, Montpellier, 34295 France; 3 Département d’Hépato-gastroentérologie A, Hôpital Saint Eloi, CHU Montpellier, France; 4 INSERM U1051, Institute of Neurosciences of Montpellier, Montpellier, France; 5 Laboratory of Hematology-Oncology, European Institute of Oncology, Milan, Italy; 6 Department of Biochemistry and Molecular and Cellular Biology, Aragón Health Research Institute (IIS Aragón), University of Zaragoza, Zaragoza, Spain; 7 Departamento de Biología Molecular, Centro de Biología Molecular Severo Ochoa, CSIC-UAM, CIBERER, Universidad autónoma de Madrid, 28049 Madrid, Spain; 8 Centro Nacional de Investigaciones Cardiovasculares Carlos III (CNIC) Melchor Fernandez Almalgo, 3 28209 Madrid, Spain; 9 CIBERFES. Melchor Fernandez Almagro, 3 28209 Madrid, Spain; 10 Département d’Hématologie Clinique, CHU Montpellier, Université Montpellier I, 80 avenue Augustin Fliche, 34295 Montpellier, France; 11 IGMM, CNRS, Univ. Montpellier, Montpellier, France; 12 Institut de Biologie Computationnelle, Montpellier, France; 13 Department of Biology and Health Sciences, University of Montpellier, Montpellier, France

## Abstract

Oxidative phosphorylation (OXPHOS) generates ROS as a byproduct of mitochondrial complex I activity. ROS-detoxifying enzymes are made available through the activation of their antioxidant response elements (ARE) in their gene promoters. NRF2 binds to AREs and induces this anti-oxidant response. We show that cells from multiple origins performing OXPHOS induced NRF2 expression and its transcriptional activity. The *NRF2* promoter contains MEF2 binding sites and the MAPK ERK5 induced MEF2-dependent NRF2 expression. Blocking OXPHOS in a mouse model decreased *Erk5* and *Nrf2* expression. Furthermore, fibroblasts derived from patients with mitochondrial disorders also showed low expression of *ERK5* and *NRF2* mRNAs. Notably, in cells lacking functional mitochondrial complex I activity OXPHOS did not induce ERK5 expression and failed to generate this anti-oxidant response. Complex I activity induces ERK5 expression through fumarate accumulation. Eukaryotic cells have evolved a genetic program to prevent oxidative stress directly linked to OXPHOS and not requiring ROS.

## Introduction

Energy consumption in organisms should be finely regulated to spare resources. The vast majority of eukaryotic cells perform oxidative phosphorylation (OXPHOS), which uses the energy generated by mitochondrial oxidation to produce adenosine triphosphate (ATP). This metabolic pathway is highly efficient in releasing energy but it produces reactive oxygen species (ROS) as a byproduct. ROS are involved in normal cell signaling and homeostasis. However, under stress conditions levels may rapidly increase resulting in cell damage, a process known as oxidative stress. Hence, cells using mitochondria as first energy source must regulate ROS levels. Logically, ROS and mitochondria are functionally linked in several ways. First, ROS in the short-term regulate mitochondrial morphology and function via non-transcriptional pathways^1^. Second, ROS lead to Kelch-like ECH-associated protein 1 (KEAP-1) degradation, thereby activating nuclear factor (erythroid-derived 2)-like 2 (NFE2L2 or NRF2)^2,3^, which regulates expression of mitochondrial genes^4^. In addition, NRF2 controls ROS production by mitochondria^5^ and mitochondrial function^6,7^. NRF2 arguably mediates the strongest anti-oxidant cellular response by binding to anti-oxidant response elements (ARE) in gene promoters and, consequently, regulates oxidative stress^2,3^. On the other hand, mitochondrial activity induced by acute exercise promotes Ref1/Nrf2 signaling and increases mitochondrial antioxidant activity and capacity in myocardial and skeletal muscle^8,9^. Remarkably, restraining OXPHOS *in vivo* in the liver strongly decreases Nrf2 levels^10^. Moreover, tumor cells forced to perform OXPHOS generate a NRF2-mediated anti-ROS response^11^. However, how mitochondria transcriptionally signal the genetic program to block the ROS they produce remains unknown.

NRF2 activation depends on its dissociation from the repressor protein KEAP1 and its subsequent translocation into the nucleus^2^. In hematopoietic cells, the MAPK extracellular signal-regulated kinase-5 (ERK5), through the transcription factor MEF2, induces expression of miR-23 that inhibits *KEAP-1* mRNA leading to NRF2 activation^11^. Several types of oxidative stress activate ERK5^12^, notably in leukemic cells^11,13,14^. In fact, ERK5 is considered a redox MAPK^15^. In endothelial cells, steady laminar blood flow (s-flow) activates ERK5 that induces up-regulation of NRF2-dependent gene expression, although the mechanism is not fully elucidated^16,17^.

Growing evidence indicates that there are alternative pathways leading to *de novo* production of NRF2^3^. In this context, KEAP-1 inhibition only partially accounts for OXPHOS-induced antioxidant response^11^. Chip-seq experiments performed by the ENCODE consortium have shown that the NRF2 promoter contains MEF2 binding sites^18^. Moreover, predicted networks of transcription factor interactions in skeletal muscle unveil direct regulation of NRF2 by MEF2A^19^ and MEF2D binds and activates the *Nrf2* promoter^20^. Hence, ERK5 could transcriptionally induce NRF2 expression through MEF2, a transcription factor that mediates some of the metabolic effects of ERK5^11,13,14,21–24^. In fact, ERK5 regulates the choice of catabolic substrates in hematopoietic cells^11,13,14,21–23^, suggesting that is a good candidate to mediate the link between OXPHOS and the antioxidant response. We hypothesize that mitochondrial activity triggers the ERK5 pathway that, through MEF2, induces NRF2 expression and NRF2-mediated antioxidant response. We validate this by showing that mitochondrial complex I activity and fumarate accumulation induce the transcriptional expression of *ERK5*. ERK5 through MEF2 induces *NRF2 de novo* expression. Therefore, mitochondrial activity is directly linked to the most important antioxidant response in the absence of *de novo* increase in ROS levels. This implies that eukaryotic cells have evolved a genetic program to prevent oxidative stress directly linked to OXPHOS and not requiring ROS.

## Results

### OXPHOS-induced de novo expression of NRF2

**Figure 1:**
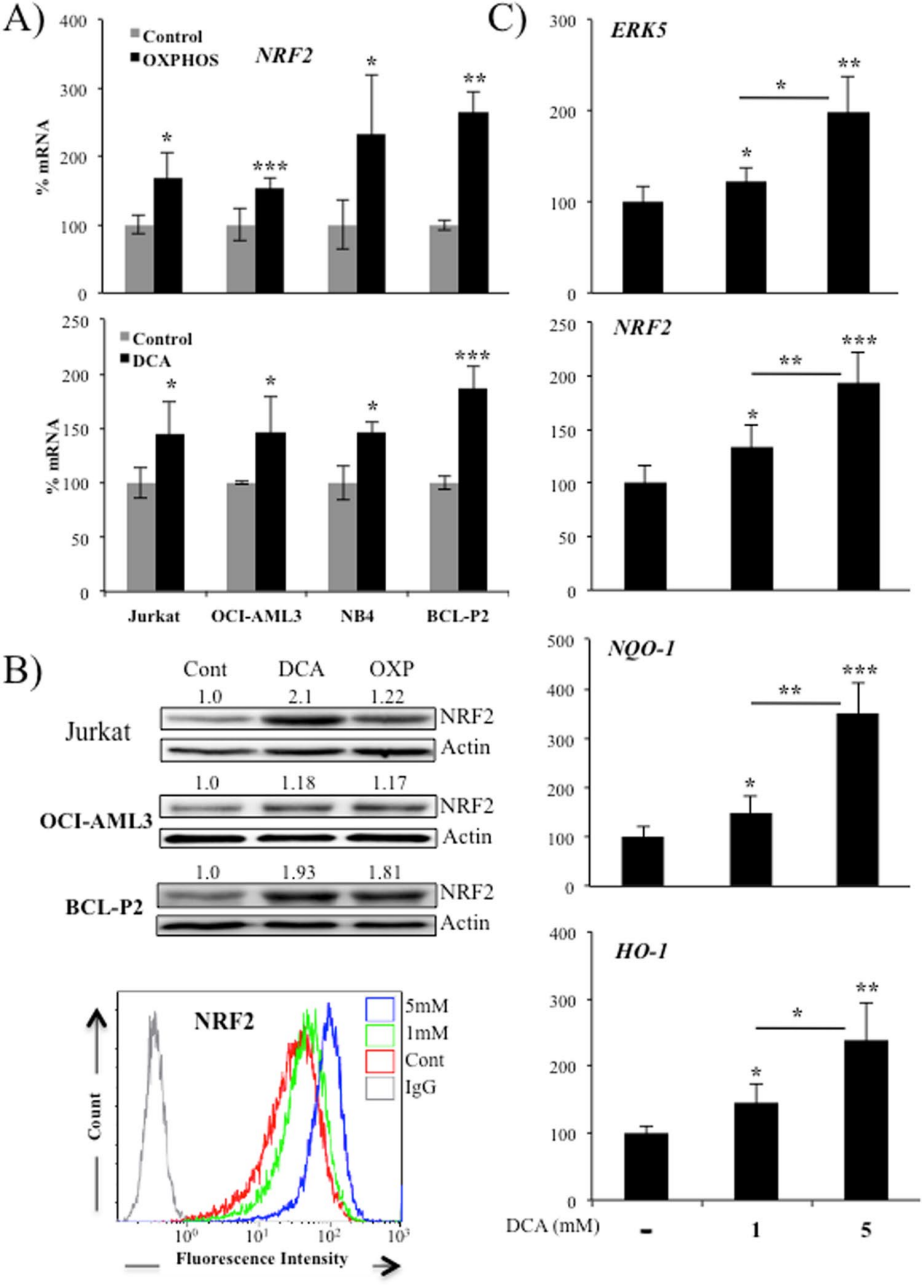
Cells performing OXPHOS upregulated NRF2 expression. (**A**) Different hematopoietic cells were incubated in OXPHOS medium or treated with 5 mM DCA for 2 weeks. *NRF2* mRNA was quantified by qPCR and values normalized to *β-actin* mRNA. Results were represented as the % of mRNA compared to cells growing only in glucose medium. Bars show average ± SD of 3 independent experiments performed in triplicate. (**B**) NRF2 protein expression was analyzed in several cell lines by western blotting (upper panel) or by flow cytometry in OCI-AML3 cells. (**C**) Hepatocytes from 4 donors were treated with the indicated concentration of DCA for 24 h and *ERK5*, *NRF2*, *NQO-1* and *HO-1* mRNA were analyzed. Bars show average ± SD of the four donors performed in duplicate.

We have previously described that leukemic cells performing OXPHOS generated an anti-oxidant response independently of ROS^11^. This response was partially mediated by an ERK5-induced increase in miR-23 that impairs expression of *KEAP-1*^11^. In parallel experiments, we found that *NRF2* mRNA was also increased in three hematopoietic cell lines and in primary cells obtained from a B-cell lymphoma (BCL) patient growing in OXPHOS medium (Fig. 1A). This glucose-free culture medium has final concentrations of 4 mM glutamine and 10 mM galactose. Glutamine is used to drive mitochondria to utilize OXPHOS and galactose allows cells to synthesize nucleic acids through the pentose phosphate pathway^13,14,25,26^. We called it ‘OXPHOS medium’, because it forced leukemic cells to use OXPHOS as primary ATP producer^13,24,27^. The PDK1 inhibitor dichloroacetate (DCA), which stimulates OXPHOS in all tested leukemic cells^11,13,14,22,27,28^, also increased *NRF2* mRNA (Fig. 1A). Both ways to stimulate OXPHOS also induced NRF2 protein (Fig. 1B). The effect of DCA on *NRF2* mRNA and protein is reproduced in two hepatic cell lines (Supplemental Fig. 1A) and in a group of primary leukemic cells from 4 patients (Supplemental Fig. 1B). Of relevance, we observed that in primary human hepatocytes DCA also increased *ERK5* and *NRF2* mRNA as well as that of the NRF2 targets *HO-1* and *NQO-1* (Fig. 1C). In summary OXPHOS induced expression of NRF2 in multiple cell contexts.

### OXPHOS induced NRF2 translocation to the nucleus

**Figure 2:**
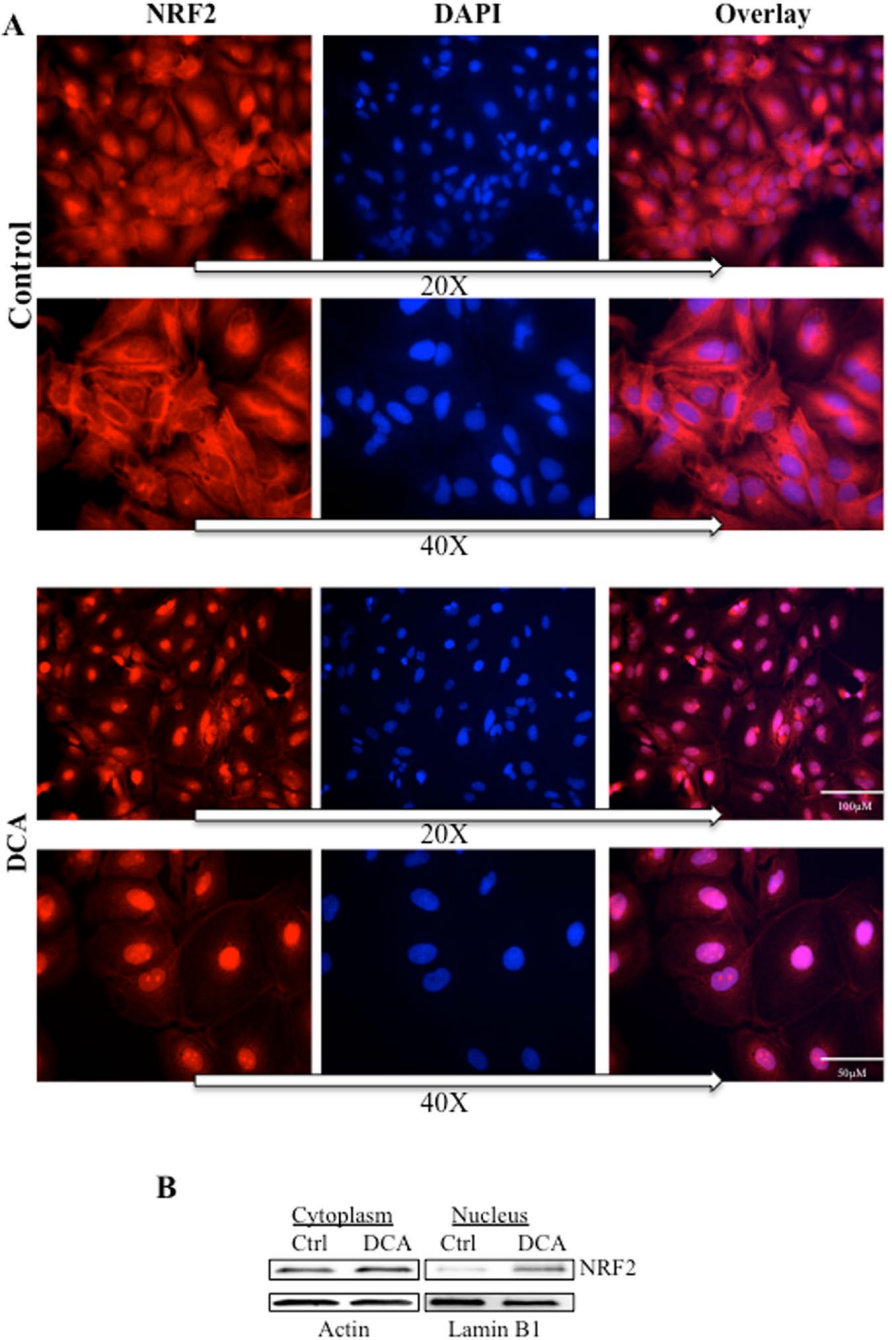
OXPHOS induced NRF2 translocation into the nucleus. (**A**) Huh7 cells were treated with 10 mM DCA for 48 h and nuclear translocation was revealed by immunofluorescence. (**B**) Jurkat cells were treated with 10 mM DCA for 48 h and NRF2 nuclear translocation was revealed by subcellular fractionation and western blotting.

NRF2 must translocate to the nucleus to activate its target genes and generate the antioxidant response. HuH7 hepatic cells treated with DCA showed NRF2 accumulation in the nucleus (Fig. 2A). These results were reproduced in non-adherent Jurkat cells by western blotting (Fig. 2B) and in the hepatic cell line HepG2C3A (Supplemental Fig. 2A). We observed a total increase in NRF2 that was more predominant in the nuclear fraction. OXPHOS medium also induced NRF2 translocation to the nucleus in Jurkat cells (Supplemental Fig. 2B).

### OXPHOS induced *de novo* expression of *NRF2 in vivo*

**Figure 3:**
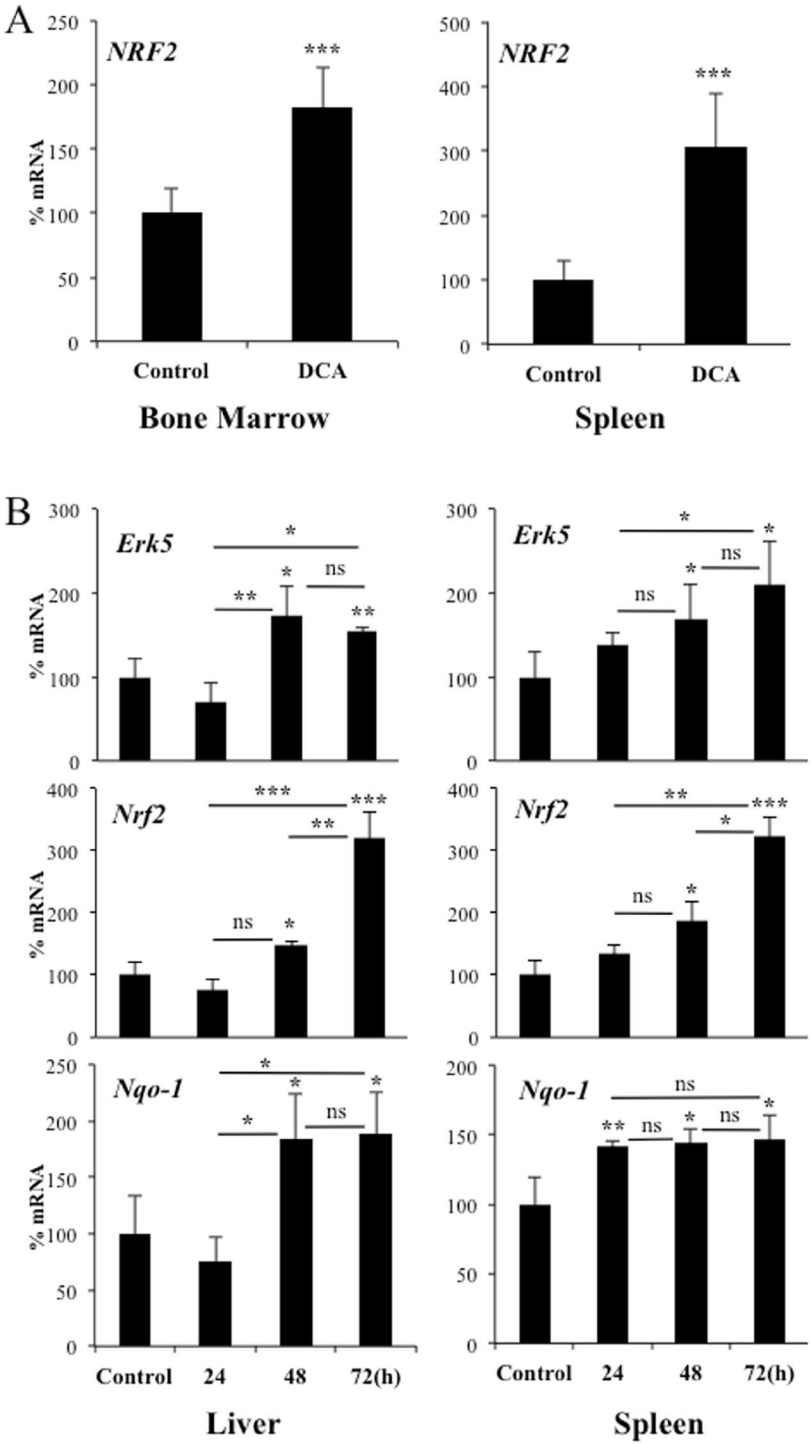
Cells performing OXPHOS induce NRF2 expression *in vivo*. (**A**) NSG mice were engrafted with primary human AML cells. At day 80 post-graft, they were treated with DCA (n = 4) or leave untreated (n = 4). At day 140, mRNA from bone marrow or spleen was isolated and the expression of different human mRNA was quantified by qPCR. (**B**) B6 wt mice (n = 4/5 per group) were treated with a dose of DCA (50 mg/kg) everyday intraperitoneally and mouse *Erk5*, *Nrf2* and *Nqo-1* mRNA was analyzed in spleen and liver at different times. The data represent means ± SD; statistics were performed using student t-test (**A**) or One-way ANOVA with post-hoc Tukey test (**B**); *p < 0.05, **p < 0.01, ***p < 0.001. Different times posttreatment were compared to non-treated mice (control) if not specified in the graph.

To test if enhanced OXPHOS could exert a similar effect on *NRF2* expression *in vivo*, we engrafted AML primary cells in non-obese diabetic/severe combined immunodeficient (NOD/SCID)-interleukin-2 receptor γ null (NSG) mice, as previously described^27^. Mice with established tumors (day 80 post-graft) were treated with DCA. The treatment was not toxic and did not show any notable effect on mouse survival^27^. Human tumor AML cells gather in mouse spleen and bone marrow, hence we isolated mRNA from these organs. We used human-specific primers to analyze the expression of the selected mRNAs and found an increase in *NRF2* mRNA (Fig. 3A). This increase paralleled that of *ER*K5 and *NQO-1* under similar conditions^11^.

DCA also induced mouse *Erk5*, *Nrf2* and *Nqo1* mRNA in liver and spleen in a separate experiment in which C57BL/6 wild type mice were treated for different periods of time, 1 to 3 days, with DCA (Fig. 3B). The effect was first observed in spleen and later in liver tissue. Nrf2 was likely active because we observed an increase in its target gene *Nqo-1* (Fig. 3B). Hence DCA induced NRF2 expression in multiple cell populations *in vitro* and *in vivo*.

### Increase in ROS levels is not essential for NRF2 expression

**Figure 4:**
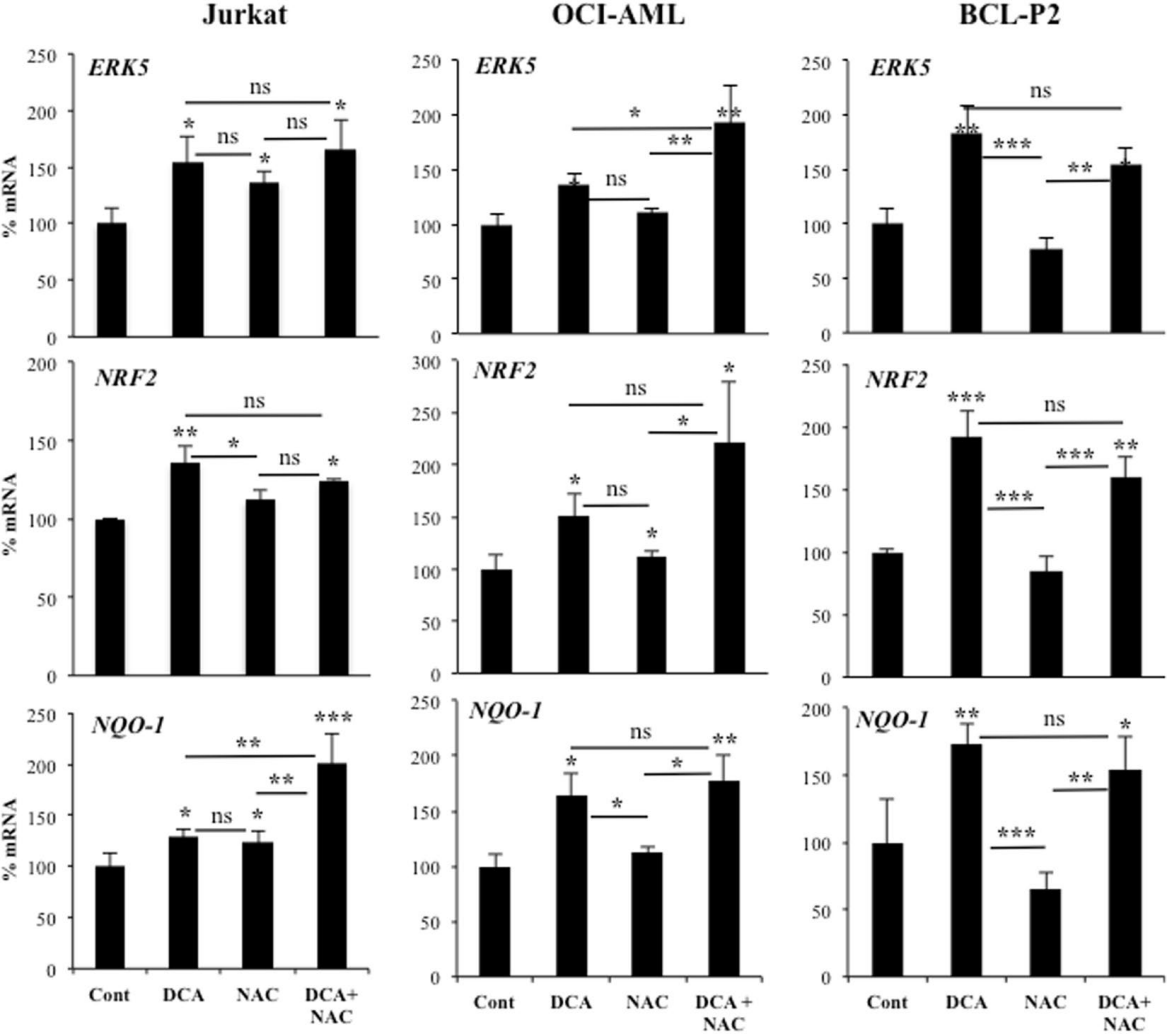
Increase in ROS levels is not required for NRF2 expression. OCI-AML and HuH7 cell lines and primary leukemic cells from a BCL patient were treated with 2 mM NAC 1 h before adding DCA (10 mM) for 24 h. mRNA was analyzed as described in Fig. 1. Experiments were done in triplicate and data represent means ± SD; statistics were performed using One-way ANOVA with post-hoc Tukey test; *p < 0.05, **p < 0.01, ***p < 0.001. Treatments were compared to non-treated cells (control) if not specified in the graph.

The cellular oxidative state can regulate NRF2 expression^2^. Therefore, we investigated whether NRF2 expression is regulated by ROS in our setting. DCA induces ROS production in some hematopoietic cell lines, e.g. OCI-AML3, but not all, e.g. Jurkat^11,27,29^. In contrast, both cell lines increased *NRF2* expression suggesting that ROS production was not essential for this induction (Fig. 4). Next, we incubated both cell lines with the antioxidant N-acetyl-cysteine (NAC), which failed to consistently reduce DCA-induced *ERK5*, *NRF2* or *NQO-1* mRNA (Fig. 4), although efficiently blocked DCA-induced ROS increase^11,29^. We observed similar results in primary leukemic cells from a BCL patient (BCL-P2). DCA does not increase ROS in the hepatic cell line HepG2C3A, but it did in Huh7^29^. However, DCA significantly increased *ERK5*, *NRF2* or *NQO-1* mRNA in both cell lines and in the presence of NAC (Supplemental Fig. 3). These results excluded a major role of ROS in NRF2 expression after DCA treatment. Normally ROS activate NRF2. Unexpectedly in AML cells, there is no relationship between high ROS levels and high nuclear NRF2^30^. Furthermore, the use of NAC, which successfully sequesters endogenous ROS in AML, has no effect on nuclear NRF2 levels^30^. Taken together, this excludes ROS as causing nuclear accumulation of NRF2 in resting human AML cells^30^.

### ERK5/MEF2 controls NRF2 expression

**Figure 5:**
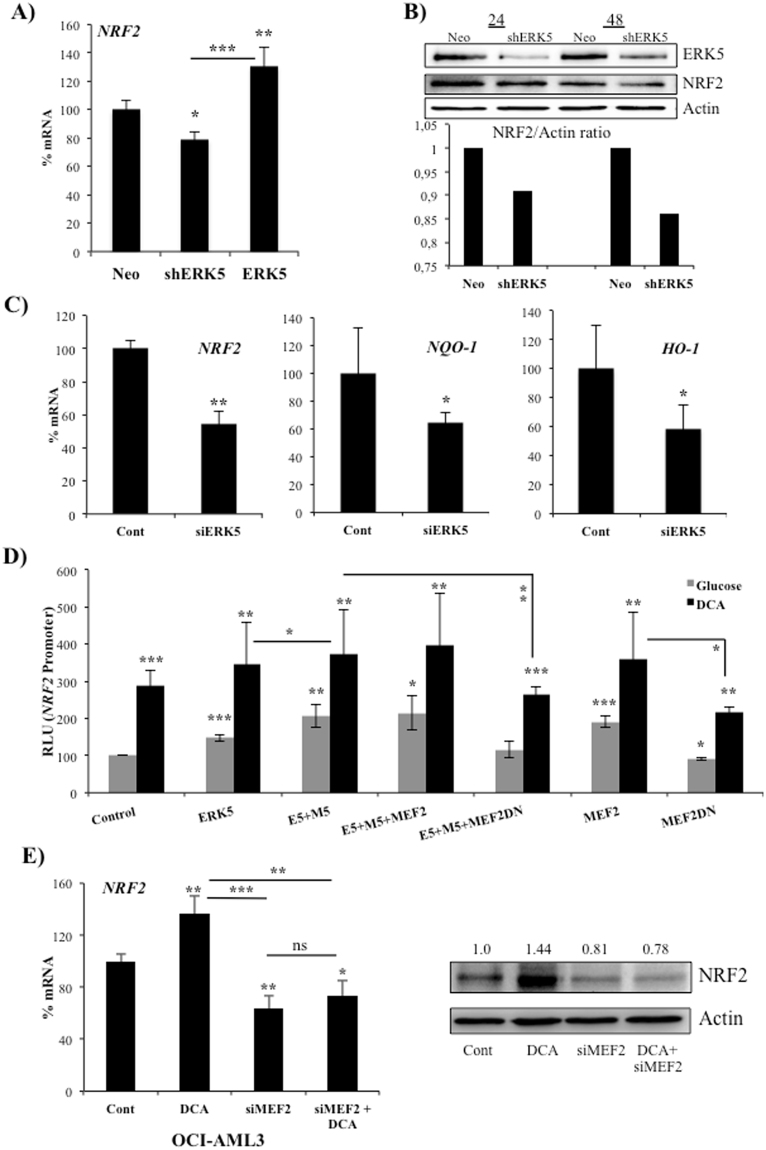
ERK5 controls NRF2 expression. (**A**) 10^7^ Jurkat-TAg cells were transfected with 5 µg of the empty pSUPER Neo vector or with this vector containing a small hairpin RNA for ERK5 (shERK5) or with a pcDNA vector expressing ERK5. Forty-eight hours later mRNA expression was analyzed by qPCR and represented as the % of mRNA compared to cells transfected with the control vector. (**B**) Cell transfected with control (Neo) or shERK5 were analyzed for protein expression by western blotting at 24 and 48 h post-transfection. Graphic bars show the NRF2/actin ratio of the depicted experiment. (**C**) Primary human hepatocytes were double transfected with control siRNA or with siRNA against ERK5 (siERK5). 96 h later mRNA was collected and mRNA expression was analyzed by qPCR. (**D**) 10^7^ Jurkat-TAg cells were co-transfected with 5 μg of the following vectors ERK5 wild type, a constitutively active MEK5 mutant (MEK5D, M5), MEF2C and MEF2C with dominant negative function (MEF2DN) together with 2 μg of a luciferase reporter plasmid driven by the *NRF2* promoter along with 1 μg of β-galactosidase expression vector. Cells were incubated in regular glucose media (gray bars) or containing 10 mM DCA (black bars) 24 h after transfection and analyzed 2 days later for luciferase and β-galactosidase activities. The graphic represents the relative luciferase units (RLU). (**E**) OCI-AML3 cells were transfected with siRNA for MEF2A and C and 24 h later treated with 10 mM DCA for 36 h. *NRF2* mRNA and NRF2 protein were analyzed as in in (**A**) and (**B**) respectively. Experiments were done in triplicate. The data represent means ± SD; statistics were performed using student t-test (**C**) or One-way ANOVA with post-hoc Tukey test (**A**,**D** and **E**); *p < 0.05, **p < 0.01, ***p < 0.001. Treatments were compared to empty vector transfected cells (control) if not specified in the graph.

Next, we investigated the mechanism responsible for mitochondria activity-induced NRF2 expression. Reducing expression of ERK5 with a small hairpin RNA (shERK5) diminished *NRF2* mRNA expression in hematopoietic cells under resting conditions (Fig. 5A). NRF2 protein levels were also reduced after shERK5 transfection (Fig. 5B). We could not treat shERK5-expressing cells with DCA because they die due to lack of appropriate mitochondrial functions and antioxidant response^11,13,14,21–23^. Conversely, overexpression of ERK5 increased *NRF2* mRNA (Fig. 5A). Reducing expression of ERK5 in primary human hepatocytes (Fig. 5C) and hepatic cell lines, HuH7 and HepG2C3A (Supplemental Fig. 1A,B), with small interference RNA for ERK5 (siERK5) also impaired expression of *NRF2* and its target genes *NQO-1* and *HO-1*.

To further study the role of the ERK5/MEF2 pathway in NRF2 expression, we overexpressed several proteins of this pathway. Strong activation of the ERK5 pathway by co-overexpression of a constitutively active mutant of MEK5 (MEK5D), the upstream kinase of ERK5, and ERK5 induced a greater increase in *NRF2* mRNA (Supplemental Fig. 4C). In those experiments, only 30–60% of the cells are effectively transfected. To overcome this issue, we use a luciferase reporter plasmid driven by a DNA fragment of 1.5 kb of the human *NRF2* promoter^30^. In this context, cells expressing the reporter plasmid also contain the overexpressed proteins. ERK5 significantly activated the reporter and MEK5D increased this effect (Fig. 5D). Expression of a dominant negative form of MEF2C (MEF2C-DN) decreased the effect of ERK5 and MEK5D. This DN construct also diminished basal or DCA-stimulated reporter expression. In contrast, MEF2C overexpression increased both basal and DCA-induced activity (Fig. 5D). DCA, which induced strong activation, did not show a synergistic, but rather an additive, effect with the activating proteins. These results suggested that ERK5 controls NRF2 expression through MEF2. To test this, we transfected a small interference RNA for MEF2 (siMEF2) in the hepatic cell line HepG2C3A (Supplemental Fig. 4D) and the AML cell line OCI-AML3 (Fig. 5E). This efficiently decreased *MEF2* mRNA and protein^29^ and also decreased both basal and DCA-induced *NRF2* mRNA (Fig. 5E).

### Mitochondrial complex I activity signals ERK5 expression

**Figure 6:**
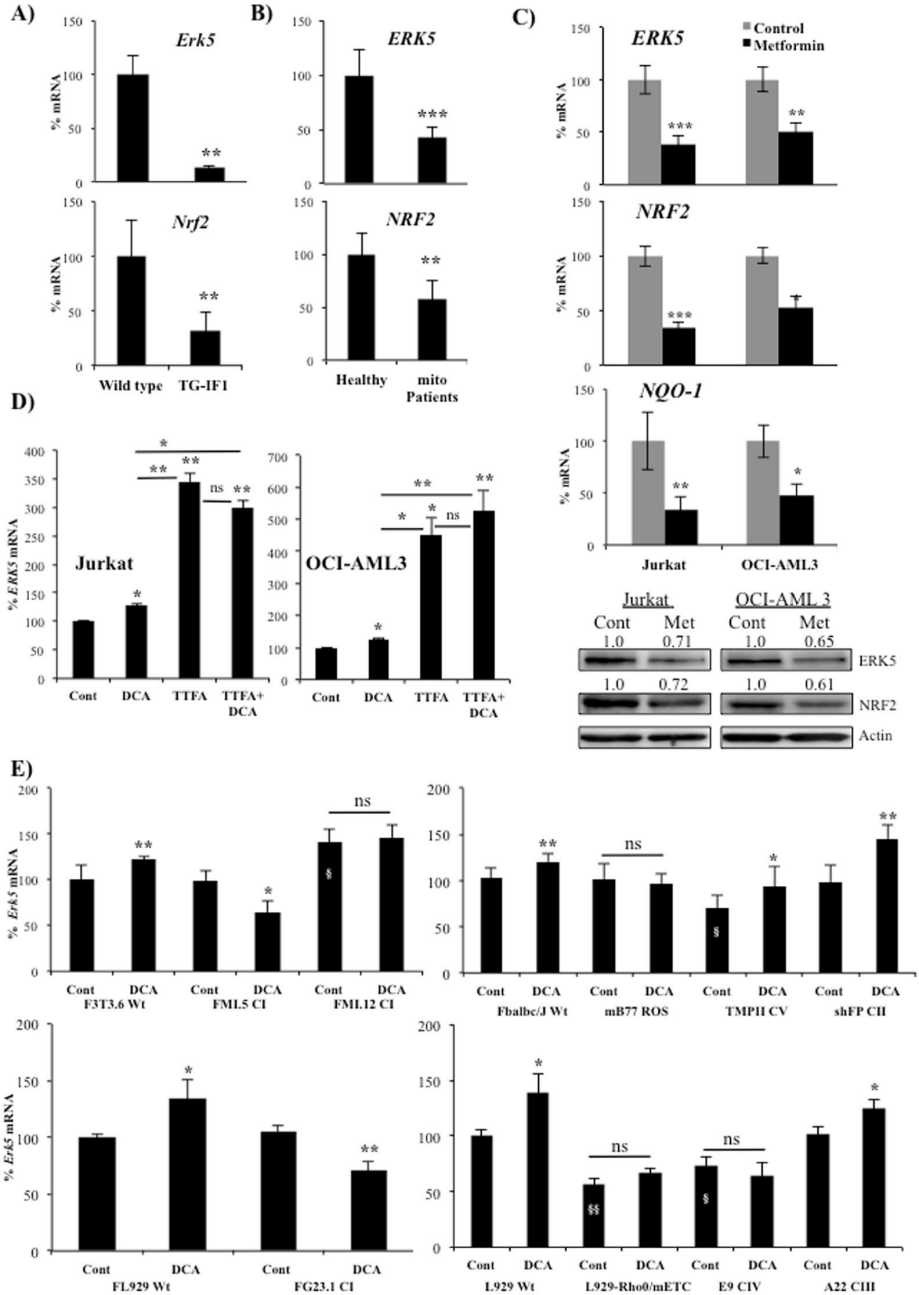
Inhibition of mitochondrial complex I and II signals ERK5 expression. (**A**) *Erk5* and *Nrf2* mRNA expression in the liver of wild-type and T/H (Tet-Off-H49K (h-IF1) mice. mRNA from 3 mice of each genotype was quantified by qPCR and represented as the % of mRNA compared to wild-type mice. (**B**) *ERK5* and *NRF2* mRNA expression in fibroblasts derived from a group of 8 healthy donors or 8 patients suffering from mitochondrial defects (Supplemental Table 1). (**C**) Different hematopoietic cell lines were incubated for 24 h with 5 mM metformin. mRNA expression was quantified by qPCR and represented as the % of mRNA compared non-treated cells. ERK5 and NRF2 protein expression was analyzed in these cell lines by western blotting (lower panel). (**D**) Jurkat and OCI-AML3 cells were treated with 10 mM DCA and 300 µM TTFA for 24 h. *NRF2* mRNA expression was quantified by qPCR and represented as the % of mRNA compared to control cells. (**E**) Different cell lines described in Supplemental Table 2 were treated with 20 mM DCA during 24 hours. *ERK5* mRNA was quantified by qPCR and represented as the % of mRNA compared to non-mutant control cells. Experiments were done in triplicate and data represent means ± SD; statistics were performed using student t-test (**A**–**C**) or One-way ANOVA with post-hoc Tukey test (**D** and **E**); *p < 0.05, **p < 0.01, ***p < 0.001; ^§^p < 0.05, ^§§^p < 0.01 compare to the respective control cell lines. Treatments were compared to non-treated cells (control) if not specified in the graph.

The previous experiments had shown that OXPHOS generates a signal that induces ERK5 expression, which contributes to the NRF2-mediated antioxidant response. We confirmed this *in vivo* by using a transgenic Tet-Off mouse that express a mutant active form of the ATPase Inhibitory Factor 1 (IF1) in hepatocytes to restrain OXPHOS in the liver^10^. Interestingly Santacatterina *et al*. describe in the Fig. 8C of their MS that liver Nrf2 levels are lower in mice expressing IF1. We confirmed it by analyzing expression of *Nrf2* mRNA (Fig. 6A). This correlated with lower expression of *Erk5* mRNA as compared to wild type mice (Fig. 6A). This shows that OXPHOS also induces *Erk5* mRNA expression *in vivo*.

We confirmed the essential role of mitochondrial function on *ERK5* expression by using fibroblasts derived from patients with strong mitochondrial disorders (Supplemental Table 1). *ERK5* and *NRF2* mRNA were significantly reduced in these patients (Fig. 6B).

We next focused on the molecular mechanisms underlying our observations. When we inhibited the mitochondrial complex I with metformin, we observed a decrease on *ERK5*, *NRF2* and *NQO-1* mRNA and protein expression (Fig. 6C). Both metformin and DCA induce AMPK activation^27^, however, they blocked and induced *ERK5* expression, respectively. This suggested that AMPK and its associated metabolic changes were not involved in ERK5 expression. We confirmed this by reducing the expression of the catalytic subunit of AMPK, AMPKα, with 2 different siRNA that effectively blocked several AMPK-mediated metabolic changes^27^. This did not affect expression of *ERK*5 or *NRF2* mRNA (Supplemental Fig. 5). Therefore, AMPK activation was not responsible for generating the antioxidant response in cells performing OXPHOS.

In Fig. 6C we showed that complex I inhibition decreased *ERK5* mRNA. The electron transport chain complex III removes electrons from ubiquinol (QH_2_) and sequentially transfer them to cytochrome c. The reduction of ubiquinone (Q) to QH_2_ could either be due to mitochondrial complex I, which removed electrons from NADH, or mitochondrial complex II, which removed them from succinate and transferred through FAD. Then, we investigated the effect of the complex II inhibitor thenoyltrifluoroacetone (TTFA). This drug strongly induced *ERK5* mRNA expression (Fig. 6D). DCA did not increase TTFA effects suggesting that both shared the same target.

TTFA was slightly toxic (Supplemental Fig. 6A) and, like metformin, could have off-target effects. Therefore, we used an array of cell lines with impaired activity of the different mitochondrial complexes (Supplemental table 2). ρ0 cells that lack mitochondrial DNA and thus a functional ETC, did not induce ERK5 expression after DCA treatment (Fig. 6E, right lower panel), in agreement with our previous results showing that mitochondrial activity induced ERK5 expression (Fig. 6A,B^13^. We next used 3 different cell lines in 2 different mitochondrial backgrounds with defects in mitochondrial complex I and observed that DCA treatment did not induce ERK5 expression (Fig. 6E, left panels). In agreement with Fig. 6D, mutation in mitochondrial complex II did not inhibit DCA-induced ERK5 expression (Fig. 6E, top right panel). Cells with mutations in complex III and V, but not in complex IV, increased ERK5 expression after DCA treatment (Fig. 6E, right panels). However, complex V mutant show lower basal *ERK5* mRNA levels in agreement with *in vivo* experiments (Fig. 6A). Mutation in the mitochondrial tRNA Ile in the L929 cell line (mB77), which produces more ROS^31^, did not increase *ERK5* mRNA (Fig. 6E, upper right panel). This supported our results in Fig. 4 showing that *de novo* ROS production was not involved in ERK5 expression.

**Figure 7:**
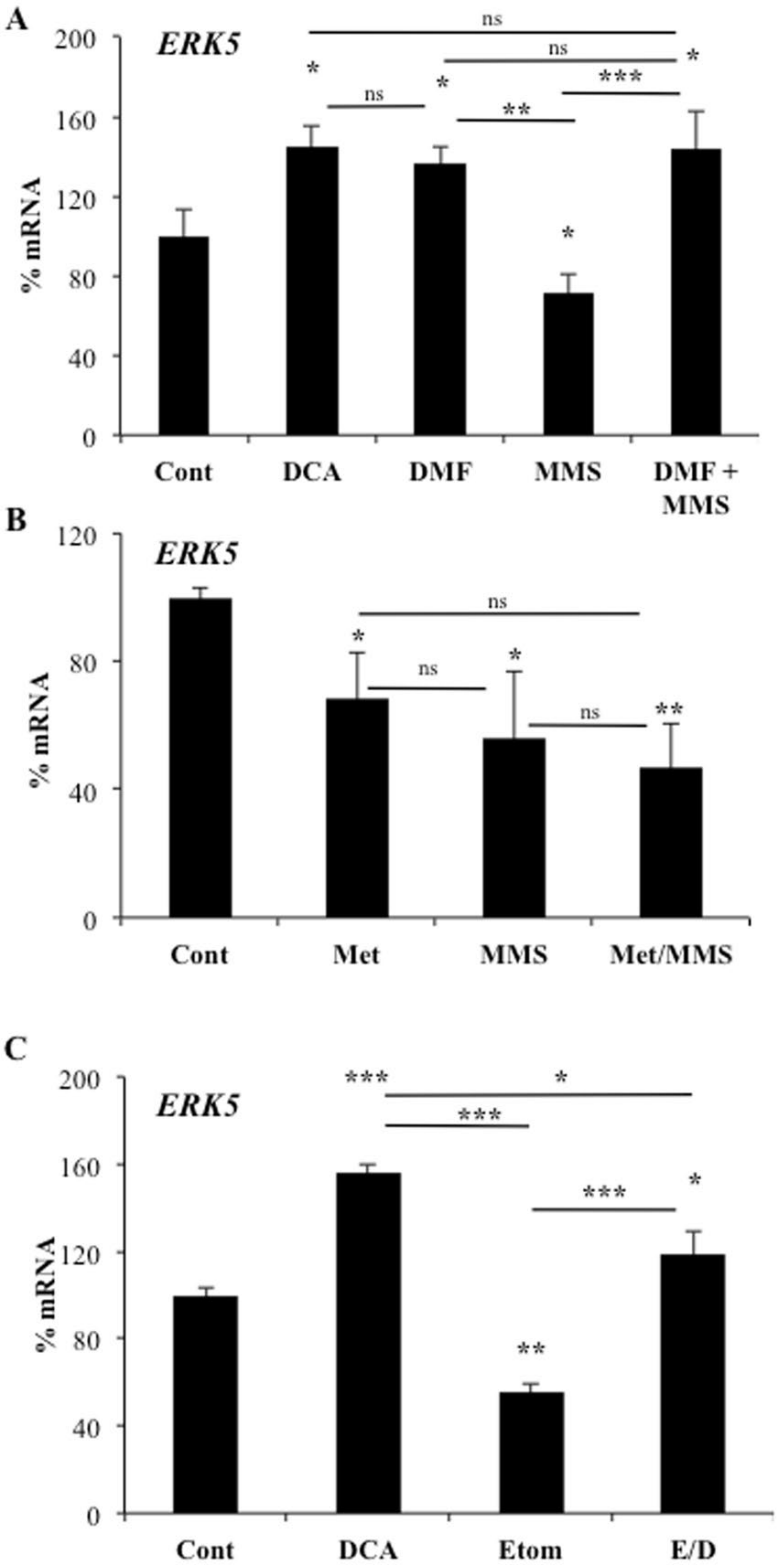
Fumarate/succinate regulate *ERK5* expression. OCI-AML3 were treated with different drug combinations and the expression of *ERK5* mRNA was analyzed by qPCR. (**A**) OCI-AML3 cells were treated with 10 mM DCA, 5 mM MMS and/or 300 µM DMF for 24 h. (**B**) OCI-AML3 cells were treated with 5 mM metformin and/or 5 mM MMS for 24 h. (**C**) OCI-AML3 cells were treated with 5 mM DCA and/or 100 µM Etomoxir for 48 h. The data represent means ± SD; *p < 0.05, **p < 0.01, ***p < 0.005 ANOVA with post-hoc Tukey test. Treatments were compared to non-treated cells (control) if not specified in the graph.

Mitochondria adapt the organization of the different complexes and supercomplexes to optimize the use of the available substrates, mainly regulating the proportion of respiratory complex III superassembled with complex I for electron transport. This is needed to avoid competition between FADH_2_- and NADH-derived electrons^31^. DCA, by inhibiting PDK1, activates PDH and the formation of acetyl-CoA from pyruvate. This generates 3 NADH per 1 FADH_2_ (through succinate) molecules in the TCA cycle. Other substrates however, generate a different proportion of NADH/FADH_2_ electrons and therefore a different demand of CI/CII dependent oxidation. Therefore, while both complexes are always delivering electrons to the ETC simultaneously, the requirement of complex I seems relatively favored by DCA. Complex II, or succinate dehydrogenase (SDH), is also part of the Krebs cycle and catalyzes the conversion of succinate to fumarate. Hence, if complex II is outcompeted by complex I activity, succinate accumulation and fumarate reduction may be induced. Both phenomena are well known in intracellular signaling. Fumarate and succinate, in their acid form, acidify culture media. Hence, we used monomethylsuccinate (MMS) and dimethylfumarate (DMF) to investigate the impact of fumarate and succinate accumulation on ERK5 expression. MMS decreased *ERK5* levels (Fig. 7A). In contrast, DMF increased them (Fig. 7A). Next, we used metformin to inhibit complex I, forcing the use of complex II, and added MMS to increase complex II activity. When used together, they decreased even further ERK5 expression (Fig. 7B). This suggested that complex II activity reduced ERK5 expression, probably by inhibiting complex I activity. In summary, whereas succinate probably does not play any role *per se* on *ERK5* expression, fumarate induces its expression. This suggested that DCA, by accumulating fumarate, induces *ERK5* mRNA.

Complex I receive electrons from NADH and deliver them to CoQ. Complex II employs FADH as co-factor to deliver electrons from succinate to CoQ.FADH_2_. Therefore, the ratio of NADH/FADH_2_ electrons changes with different substrates and the requirement of complex I for NADH oxidation vary according to the NADH/FADH_2_ ratio. Oxidative metabolism of one molecule of glucose generates ten NADH and two FADH2, a NADH:FADH2 electron ratio of 5. Fatty acids (FA), e.g. palmitate, generate a ratio of 2^32^. Etomoxir inhibits FA transport into the mitochondria and blocks fatty acid oxidation (FAO), resulting in an increase of the ratio NADH/FADH_2_. Interestingly, etomoxir decreased basal and DCA-induced increase *ERK5* mRNA (Fig. 7C). Therefore, we found no correlation between *ERK5* expression and the expected changes in NADH/FADH_2_ ratio. Etomoxir and DCA were not toxic to OCI-AML3 cells, although they decreased cell proliferation (Supplemental Fig. 6B). However when combined they induced cell death suggesting that DCA treatment requires FAO for cell survival as suggested by our previous results^29^. In summary complex I activity, through accumulating fumarate, induces ERK5 expression leading to NRF2-mediated antioxidant response.

## Discussion

ROS generation is inherent to the activity of the electron transport chain, with Complex I being considered one of the main sites at which premature electron leakage to oxygen occurs and give rise to superoxide anion^33^. We show here that complex I activity initiates an antioxidant response mediated by ERK5-induced NRF2 expression. It is interesting to note that the main generator of ROS is at the same time responsible of triggering the mechanism to eliminate them. Of relevance, ROS *de novo* production is not required for this response. The cell “anticipates” ROS formation and activates the pathway to avoid their uncontrolled increase. Once produced, ROS quickly originate biochemical reactions that generate damage to cell structures. Hence, it is on the cell’s own benefit to create the antioxidant response when ROS production is going to occur. However, new data are challenging the “only” deleterious view of mitochondrial ROS. For example ROS increase with aging, but increasing mitochondrial ROS production specifically through the respiratory complex I reverse electron transport (RET) extends *Drosophila* lifespan^34^.

We show that mitochondrial complex I activity is required for ERK5-induced NRF2 expression. We have examined several possibilities that could account for our observation. DCA inhibits glycolysis and increases FAO as suggested by the high toxicity of the combined etomoxir plus DCA treatment ((Supplemental Fig. 6) and^29^). Then, this switch would change NADH:FADH2 electron ratio. When electron flux from FAD overwhelms the oxidation capacity of CoQFAD, CI is degraded, releasing CIII from CI-containing complexes to receive FAD-derived electrons^35^. The increased electron flux through FAD could saturate the oxidation capacity of the dedicated coenzyme Q (CoQ) pool and result in the generation of ROS^36^. However, in our experiments ROS do not mediate OXPHOS-induced *ERK5* expression (Figs 4 and 6). Moreover, etomoxir decreases basal *ERK5* expression but it does not block DCA-induced increase. Finally, if FAO is inducing ERK5 expression, complex II inhibition should decrease it, but we found an increase with TTFA and basically no effect by genetic approaches. This suggests that another mechanism is responsible for triggering ERK5 expression.

In non-transformed cells inhibition of OXPHOS by IF1 induces AMPK activation^10^, which could lead to ERK5 activation^37^. DCA also induces AMPK^27^. However, our pharmacological (metformin, Fig. 6C) and genetic (siRNA, Supplemental Fig. 5) approaches suggest that AMPK is not involved on ERK5 expression during OXPHOS.

Strong mitochondrial complex I activity could decrease electron transport through complex II and the subsequent accumulation of succinate or reduced fumarate be responsible for ERK5 expression. Our results using TTFA or genetically-modified cells support this conclusion (Fig. 6). However, MMS fails to induce ERK5 even if complex I was blocked by metformin (Fig. 7). In contrast, we found that DMF induced ERK5 (Fig. 7). Interestingly, DMF induces Nrf2 expression through a PD98059-sensitive pathway^38^. Although this MAPKK inhibitor was initially described as a specific MEK1 inhibitor, it also inhibits the ERK5 upstream kinase MEK5^39^. Therefore, fumarate indeed mediates *ERK5* expression. Accumulated fumarate can covalently modify cysteine residues of proteins, in an uncatalyzed process termed succination, modifying cellular signaling^40^. Succination occurs on KEAP1^41^ and results in constitutive NRF2 activation and increased expression of its target genes^41^. Therefore, fumarate induces the NRF2-mediated antioxidant response by directly affecting KEAP-1^41^ and by inducing *de novo* NRF2 expression (our results).

An alternative is that succinate promotes CII activity and induces RET thereby decreasing mitochondrial membrane potential. In reverse, fumarate blocks CII thereby increasing complex I activity and this could trigger ERK5 expression.

Although it is well-established that ROS induce NRF2 activation^2^, recent data support that alternative pathways independent of ROS are also operative. For example, OXPHOS decreases KEAP-1 expression independently of ROS^11^ and NRF2 expression in AML depends on NF-κB but not on ROS^30^. Interestingly, ERK5 activates NF-κB in leukemic cells^42^. Hence, ERK5 could handle the NRF2-mediated antioxidant response by at least 3 mechanisms independently of *de novo* ROS generation: i) direct transcription through MEF2 (the results presented here); ii) direct transcription through NF-κB^30,42^; iii) upregulation of miR-23 and downregulation of KEAP1 mRNA^11^. This emphasizes the central role of ERK5 in the antioxidant response^11–17^.

Transcriptome analysis shows that the ERK5 pathway regulates in normoxia several genes involved in metabolic remodeling, including some controlled by hypoxia inducible factor-1α (HIF-1α under hypoxia^13,43^. Also, like HIF-1α, ERK5 is degraded by a process depending on the tumor suppressor von Hippel-Lindau (VHL), through a prolyl hydroxylation-dependent mechanism^44^. Hence, mitochondrial complex I activity through fumarate accumulation could also protect ERK5 from VHL-induced degradation. This is based on the fact that succinate and fumarate (and succinate) outcompete α-ketoglutarate, an essential co-factor of prolyl hydroxylase domain enzymes^45^.

How ERK5 induces *NRF2* mRNA expression is not totally elucidated. ERK5 directly phosphorylates MEF2A, C and D at different serines and threonines^46,47^. It activates MEF2A and D by direct interaction because ERK5 serves as a MEF2 coactivator through its signal-dependent direct association with the MEF2 MADS domain; although, at least, MEF2A-dependent transcription requires ERK5 kinase activity^48,49^.

Finally, forcing cells to produce energy through OXPHOS also affects cell viability and proliferation independently of ROS. This is rather related to energy depletion. In this sense OXPHOS requires mitochondrial function and DCA induces cell death in ρ0 cells, while in other cells it just inhibits growth^11,23,50^. In summary forcing OXPHOS *in vitro* is cytostatic in “normal” tumor cells and cytotoxic in cells with major mitochondrial dysfunctions.

## Experimental Procedures

### Ethical statement

Experimental procedures were conducted according to the European guidelines for animal welfare (2010/63/EU). Protocols were approved by the Animal Care and Use Committee “Languedoc-Roussillon” (approval number: CEEA-LR-12163). The use of human specimens for scientific purposes was approved by the French National Ethics Committee. All methods were carried out in accordance with the approved guidelines and regulations of this committee. Written informed consent was obtained from each patient prior to surgery.

### Reagents and antibodies

DCA was from Santa Cruz Technologies. Galactose and glutamine were from GIBCO. RIPA buffer to prepare protein extracts was from Euromedex. The complete protease inhibitor cocktail (Complete EDTA-free) and the phosphatase inhibitor cocktail (PhosSTOP) were from Roche. H_2_O_2_, DMF and MMS were from Sigma. ERK5 and NRF2 antibodies were from Cell Signaling Technology and Santa Cruz respectively. The antibody against β-Actin and HRP-labeled secondary antibodies were from Sigma.

### *In vivo* mouse experiments

*In vivo* experiments were carried out using 6 to 8 weeks/old male NSG mice. Mice were bred and housed in pathogen-free conditions in the animal facility of the European Institute of Oncology–Italian Foundation for Cancer Research (FIRC), Institute of Molecular Oncology (Milan, Italy). For engraftment of human cells, 1 million AML cells were injected intravenously (i.v.) through the lateral tail vein in non-irradiated mice. NSG mice with established human AML tumors (day 80 post-graft) were treated with DCA (50 mg/kg, 1 dose/day by gavage, starting at day 1 for 16 consecutive days). Human tumor AML cells gather in mouse spleen and bone marrow, hence we isolated mRNA from these organs. We used human-specific primers to visualize expression of human mRNA. In a different experiment B6 wild type mice were treated with a daily single dose of DCA (50 mg/kg/day) intraperitoneally and mouse mRNA was analyzed in spleen and liver after different times.

### hIF1 Transgenic mice

The samples from transgenic mice containing the mutant H49K version of hIF1 have been described^10^. mRNA was analyzed in liver of these mice.

### Cell lines and culture conditions

The leukemic human cell lines T Jurkat Tag, NB4 and OCI-AML3 were grown in RPMI 1640–Glutamax (GIBCO) supplemented with 5% (Jurkat) or 10% (OCI and NB4) FBS. Primary cells from a lymphoma B cell patient (BCL-P2) were grown in the same medium with 10% FBS. In certain experiments cells were grown in RPMI 1640 without glucose (GIBCO 11879) with the addition of 2 mM glutamine and 10 mM galactose (OXPHOS medium). The Jurkat TAg cells carry the SV40 large T Ag to facilitate cell transfection. HepG2C3A and HuH7 cells were grown in MEM and DMEM respectively supplemented with 10% FBS, sodium pyruvate, glutamine, penicillin and streptomycin. The HCT116 human colon cancer cells were cultured in low glucose (5 mM) DMEM medium supplemented with 10% FBS. Cellular confluence during experiments was between 80–85%.

### Primary Leukemic Cells

Data and samples from patients with different hematological cancers were collected at the Oncology and Clinical Hematology Department of the CHU Montpellier, France, after patient’s informed consent. Patients were enrolled in two independent clinical programs approved by the “Comités de Protection des Personnes Sud Méditerranée I (ref 1324)” and ID-RCB: 2011-A00924-37. All samples from cancer patients were collected at diagnosis.

### Human liver samples and preparation of primary human hepatocytes (PHHs) cultures

PHHs were isolated as described previously^51^ from donor organs unsuitable for transplantation or from liver resections performed in adult patients for medical reasons unrelated to our research program. Liver samples were obtained from the Biological Resource Center of Montpellier University Hospital (CRB-CHUM; http://www.chu-montpellier.fr; Biobank ID: BB-0033-00031) and this study benefitted from the expertise of Dr Jeanne Ramos (hepatogastroenterology sample collection) and Prof Sylvain Lehmann (CRB-CHUM manager). The procedure was approved by the French Ethics Committee and written or oral consent was obtained from the patients or their families.

Human hepatocytes isolation and culture were performed as described previously^51^. Briefly, after liver perfusion, hepatocytes were counted and cell viability was assessed by trypan blue exclusion test. A suspension of 1 × 10^6^ cells/mL per well was added in 12-well plates pre-coated with type I collagen (Beckton Dickinson) and cells were allowed to attach for 12 h. Then, the supernatant containing dead cells and debris was removed and replaced with 1 mL of serum-free long-term culture medium (Lanford medium, LNF). The number of confluent attached cells was estimated at ~1.5 × 10^5^ cells/cm^2^.

### Plasmids

The luciferase reported plasmid driven by a DNA fragment of 1.5 kb of the human *NRF2* promoter was a kind gift from Stuart Rushworth^30^. The expression vectors for ERK5, the pSUPER expression vector for GFP alone or GFP plus shERK5 and the pSiren-retroQ-puro (BD Biosciences) retroviral vectors for shERK5 and control have been previously described^52^. Control, MEF2A and C and ERK5 siRNA were ON-TARGETplus SMARTpools (mixture of 4 siRNA) from Dharmacon.

### Transient transfection

Jurkat cells in logarithmic growth phase were transfected with the indicated amounts of plasmid by electroporation^42,53^. In each experiment, cells were transfected with the same total amount of DNA by supplementing with empty vector. Cells were incubated for 10 min at RT with the DNA mix and electroporated using the Gene Pulser Xcell™ Electroporation system (Bio-Rad) at 260 mV, 960 mF in 400 µl of RPMI 1640. Expression of the different proteins was confirmed by western blot. The transfection efficiency in Jurkat TAg cells is between 60 and 80%. OC-AML-3 cells were transfected using Amaxa ^TM^ D-Nucleofector ^TM^ Lonza Kit according to manufactured protocol. In HuH7 and HCT116 cells, transfection of 30–50 nM siRNAs was carried out using Lipofectamine RNAiMAX (Invitrogen) in Opti-MEM (Invitrogen), according to the manufacturer’s instructions. Adherent primary hepatocytes were transfected twice at day first and third post-seeding with 20 nM siRNA. Cells were harvested 48 to 96 h post-transfection.

### Reporter assay

In all experiments, Jurkat cells were transfected with β-galactosidase reporter plasmid^42,53^. The transfected cells were harvested after 2 days and centrifuged at 1000 g for 5 min. The cell pellet was suspended in 1 ml cold PBS and transferred to 1.5 ml Eppendorf tube for washing. Cells were lysed with 100 μl luciferase lysis buffer (Promega) and incubated at room temperature for 10 min. The lysates were centrifuged and luciferase assays (40 µl) performed according to the manufacturer’s instructions (Promega, Charbonnières, France) using a Berthold luminometer. For β-Galactosidase assays, 40 µl of lysates were added to 200 µl of β-Galactosidase assay buffer (50 mM phosphate buffer pH 7.4; ONPG 200 µg; 1 mM MgCl2; 50 mM β-Mercaptoethanol) and the absorbance measured at 405 nm. The results were expressed as luciferase units normalized to the corresponding β-galactosidase activity. The expression level of the transfected proteins was routinely control by immunoblot analysis.

### Subcellular fractionation

For preparation of nuclear extracts, Jurkat cells were grown in indicated medium. Ten million cells were taken and washed twice in cold PBS^54^. Nuclear and cytoplasmic proteins were extracted using according to manufactured instruction of Bio Basic Inc®. Extracted soluble proteins were analyzed by immunoblotting.

### Counting and determination of cell viability

Cell number, viability and cell death was analyzed with the Muse Cell Analyzer (Millipore) by incubating cells with Muse Count & Viability and Annexin V and Dead Cell kits respectively, following manufacturer’s instructions^27^.

### Immunofluorescent assay

Control or treated cells were washed with cold buffer and fixed with paraformaldehyde (3.2% in PBS) for 20 minutes. Cells were washed 3 times with PBS and stored at 4 °C until labelling. Cells were permeabilized with Triton (0.1% in TBS) for 5 minutes and washed with TBS-T (TBS + Tween 0.05%). Cells were labelled with primary antibody (for one hour at room temperature (dilution in TBS + 2% SVF) and washed with TBS-T. Cells were labeled with secondary antibody and Hoechst or DAPI (1/1000 dilution in TBS + 2% SVF) for 30 minutes. Cells were washed with TBS-T and finally washed with H_2_O before montage. Immunofluorescent labeling was examined under a fluorescent microscope (Leica Microsystem, Rueil-Malmaison, France) and images were analyzed using Metamorph software (Universal Imaging Corporation, Downington, PA).

### RT-PCR

Total RNA was extracted using NucleoSpin RNA isolation columns (Macherey-Nagel), reverse transcription was carried out using iScript™ cDNA Synthesis Kit (Biorad). Quantitative PCR was performed with KAPA SYBR Green qPCR SuperMix (Cliniscience) and a CFX Connect™ Real-Time qPCR machine (Biorad) with ERK5, NRF2, NQO1, HO-1 and actin primers. Supplemental Table 3 shows all primers used in this study. All samples were normalized to *β-actin* mRNA levels. Results are expressed relative to control values arbitrarily set at 100^27^.

### Immunoblotting

Protein analysis by immunoblotting was performed essentially as previously described^27^. Briefly, samples were collected, washed out with PBS and lysed with RIPA buffer. Protein concentration was determined by BCA assay (Pierce) before electrophoresis in 4–15% TGX gels (BioRad) and equal amount of protein was loaded in each well. Protein transfer was performed in TransTurbo system (BioRad) in PVDF membranes. After blocking for 1 h with 5% non-fat milk, membranes were incubated overnight at 4 °C in agitation with primary antibodies, washed three times with PBS-Tween 0,1% and incubated with the appropriate HRP-labeled secondary antibody for 1 h. Membranes were washed out three times with PBS-Tween 0,1% and developed with Substrat HRP Immobilon Western (Millipore). Band quantification was performed using the “ImageLab” software from BioRad and represented as the ratio between the protein of interest and a control protein i.e. actin. The value of 1 is arbitrarily given to control cells. One blot representative of several experiments is shown.

### Statistical analysis

The statistical analysis of the difference between means of paired samples was performed using the paired t test. Multiple comparisons were performed using One-way ANOVA with post-hoc Tukey HSD test. The results are given as the confidence interval (*p < 0.05, **p < 0.01, ***p < 0.005). All the experiments described in the figures with a quantitative analysis have been performed at least three times in duplicate. Other experiments were performed three times with similar results. We used actin as a loading control and the histograms represent the ratio (value of protein of interest)/(value of actin).

## Electronic supplementary material

Supplementary information
