# Supplementary material for: Mitochondrial Complex I activity signals antioxidant response through ERK5
Source: Sci Rep. 2018 May 9;8:7420. doi: 10.1038/s41598-018-23884-4 (PMC5943249; doi:10.1038/s41598-018-23884-4)
Supplement: Supplementary file 1 — Supplementary information [file 41598_2018_23884_MOESM1_ESM.pdf]

Mitochondrial Complex I activity signals antioxidant response through ERK5

Abrar Ul Haq Khan[1], Nerea Allende-Vega[1,2], Delphine Gitenay[1], Johan Garaude[1], Dang-Nghiem Vo[1], Sana Belkhala[1], Sabine Gerbal-Chaloin[1], Claire Gondeau[1,3], Martine Daujat[1], Cécile Delettre[4], Stefania Orecchioni[5], Giovanna Talarico[5], Francesco Bertolini[5], Alberto Anel[6], José M. Cuezva[7], Jose A. Enriquez[8,9], Guillaume Cartron[10], Charles-Henri Lecellier[11,12], Javier Hernandez[1] and Martin Villalba[1,2].

[1] IRMB, INSERM, Univ Montpellier, Montpellier, France

[2] Institut de Regenerative Medicine et Biothérapie (IRMB), CHU Montpellier, Montpellier, 34295, France.

[3] Département d'Hépato-gastroentérologie A, Hôpital Saint Eloi, CHU Montpellier, France

[4] INSERM U1051, Institute of Neurosciences of Montpellier, Montpellier, France; Department of Biology and Health Sciences, University of Montpellier, Montpellier, France.

[5] Laboratory of Hematology-Oncology, European Institute of Oncology, Milan, Italy

[6] Department of Biochemistry and Molecular and Cellular Biology, Aragón Health Research Institute (IIS Aragón), University of Zaragoza, Zaragoza , Spain.

[7] Departamento de Biología Molecular, Centro de Biología Molecular Severo Ochoa, CSIC-UAM, CIBERER, Universidad autónoma de Madrid, 28049, Madrid, Spain

[8] Centro Nacional de Investigaciones Cardiovasculares Carlos III (CNIC) Melchor Fernandez Almalgo 3 28209 Madrid, Spain

[9] CIBERFES. Melchor Fernandez Almagro 3 28209 Madrid, Spain

[10] Département d'Hématologie Clinique, CHU Montpellier, Université Montpellier I, 80 avenue Augustin Fliche, 34295 Montpellier, France.

[11] IGMM, CNRS, Univ. Montpellier, Montpellier, France

[12] Institut de Biologie Computationnelle, Montpellier, France

Patients samples with mitochondrial diseases		
**Gene**	**Description**	**Number**
WFS1	Wolfram syndrome	01
FXN	Friedreich's ataxia	01
OPA3	Optic Atrophy 3	01
mtDNA	Leber's hereditary optic neuropathy	01
RTN4IP1	Optic Atrophy-10 with or without ataxia, mental retardation and seizures	01
NDUFA13	Hypotonia, Dyskinesia and sensorial Deficiencies	01
Unidentified	Unidentified mito disease	01
OPA1	Optic Atrophy 1	01
	Healthy	08

Supplemental Table 1. List of patients suffering from various mitochondrial diseases.

Name	Nuclear Background	mtDNA Background	Mutation	Oxphos Complex
L929	L929 (C3H/An)	L929 (C3H/An)	Wt	N/A
L929-Rho0	L929 (C3H/An)	no mtDNA	no mtDNA	No mETC
E9	L929 (C3H/An)	L929 (C3H/An)	mt-COXI (T6589C)	Complex IV
A22	L929 (C3H/An)	L929 (C3H/An)	mt-Cytb (G15263A)	Complex III
FL929	L929 (C3H/An)	L929 (C3H/An)	Wt	N/A
FG-23.1	L929 (C3H/An)	L929 (C3H/An)	mt-ND6 (iC13887)	Complex I
FBalbc/J	L929 (C3H/An)	Balb/cJ	WT	N/A
mB77	L929 (C3H/An)	Balb/cJ	mt-tRNA Ile (G3739GA)	tRNA Ile, more ROS
Bc-TMP-II	L929 (C3H/An)	Balb/cJ	mt-ATP6 (A8414G)	Complex V
FBalb/cJ-ShFp	L929 (C3H/An)	Balb/cJ	ShRNA-FpSDH	Complex II
FMI.12	L929 (C3H/An)	NIH.3T3	mt-ND4 (delA10227)	Complex I
FMI.5	L929 (C3H/An)	NIH.3T3	mt-ND6 (delC13887)	Complex I
F3T3.6	L929 (C3H/An)	NIH.3T3	Wt	N/A

Supplemental Table 2. Mice fibroblasts with different mitochondrial mutations.

List of Primers	
***ERK5* (H):**	Forward: CGCTACTTCCTGTACCAACTGC<br>Reverse: AGCCATACCAAAGTCACCAATC
***NRF2* (H):**	Forward: AAA CCA CCC TGA AAG CAC AG<br>Reverse: AGT GTT CTG GTG ATG CCA CA
***HO-1* (H):**	Forward: ACA AGG AGA GCC CAG TCT TC<br>Reverse: AGA CAG GTC ACC CAG GTA GC
***NQO-1* (H):**	Forward: CCT CTA TGC CAT GAA CTT<br>Reverse: TAT AAG CCA GAA CAG ACTC
***ACTIN* (H):**	Forward: GAGGGAAATCGTGCGTGACA<br>Reverse: AATAGTGATGACCTGGCCGT
***Erk5* (M):**	Forward: TATCATGGCCATCAAGGACA<br>Reverse: AGCGGCTGTGAAGAGTGAAT
***Nrf2* (M):**	Forward: CCATTTACGGAGACCCACCGCCTG<br>Reverse: CTCGTGTGAGATGAGCCTCTAAGCGG
***Nqo-1* (M):**	Forward: GGCATCCTGCGTTTCTGTG<br>Reverse: GGTTTCCAGACGTTTCTTCCAT

Supplemental Table 3. Primers Used in this study

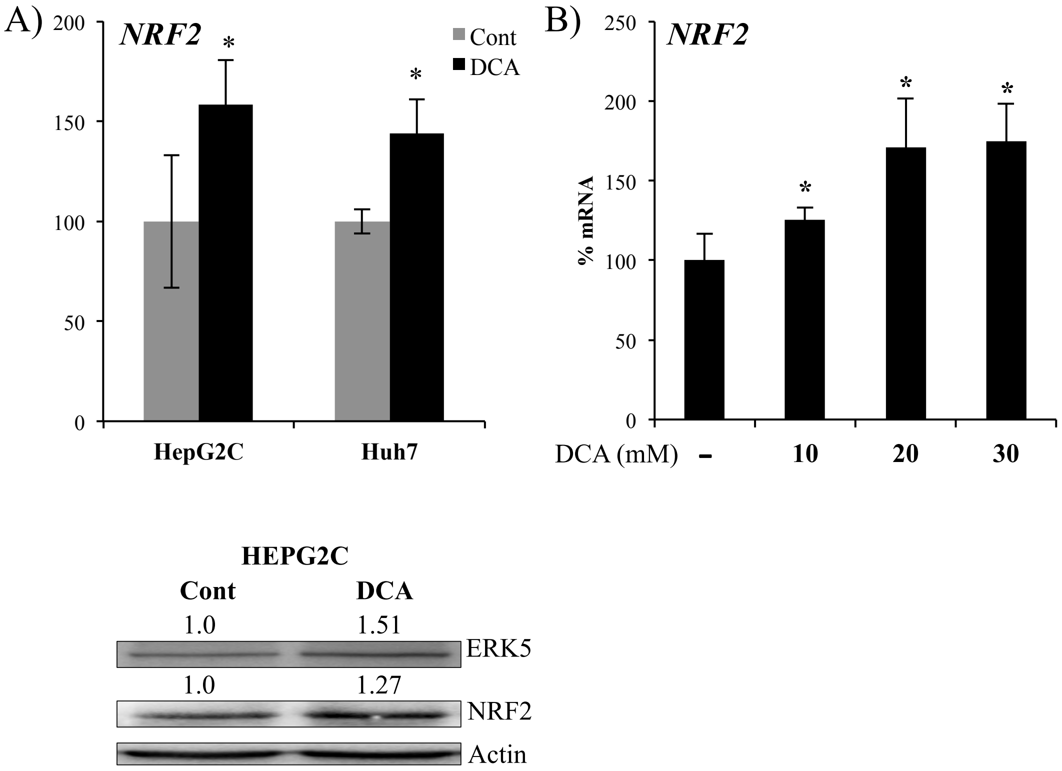

Supplemental Fig. 1. A) Hepatic Cells were were treated with 10 mM DCA for 24h and *NRF2* mRNA (upper) and protein (lower) were measured. The ratio ERK5/actin and NRF2/actin is depicted in the figure. Results represent the means ± SD of 3 independent experiments performed in triplicate. B) Tumor cells from hematological cancer patients (2 MM, 1 B-CLL and 1 T Lymphoma) were treated with various concentration of DCA for 24 h and *NRF2* mRNA was analyzed. * p<0.05, ** p<0.01, *** p<0.005 student t-test compare to non-treated cells.

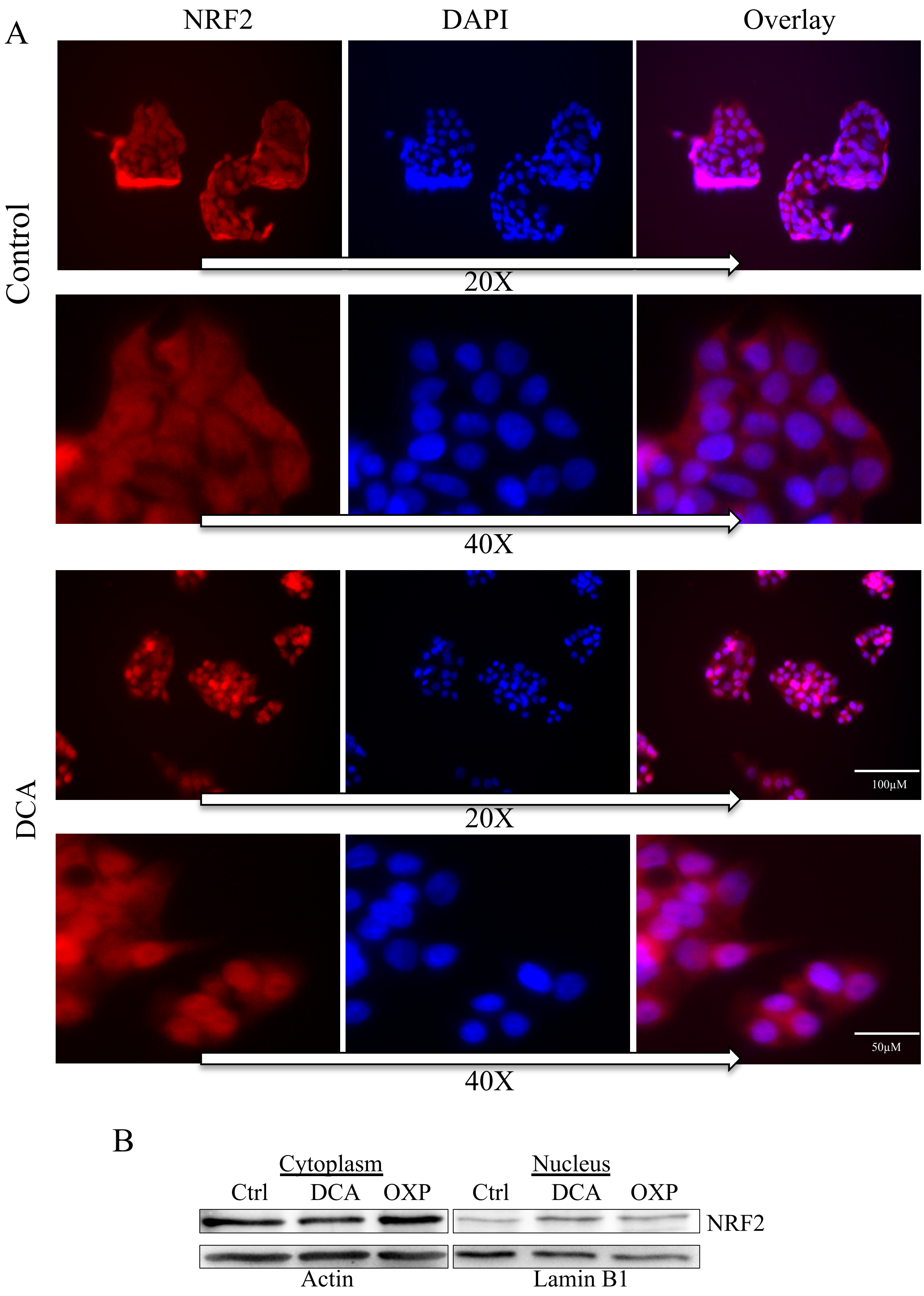

Supplemental Fig. 2. OXPHOS induced NRF2 translocation into the nucleus. A) HepG2C cells were treated with 10 mM DCA for 48 h and nuclear translocation was revealed by immunofluorescence. B) Jurkat cells were treated with 10 mM DCA for 48h or kept in OXPHOS for 1 week and NRF2 nuclear translocation was revealed by subcellular fractionation and WB.

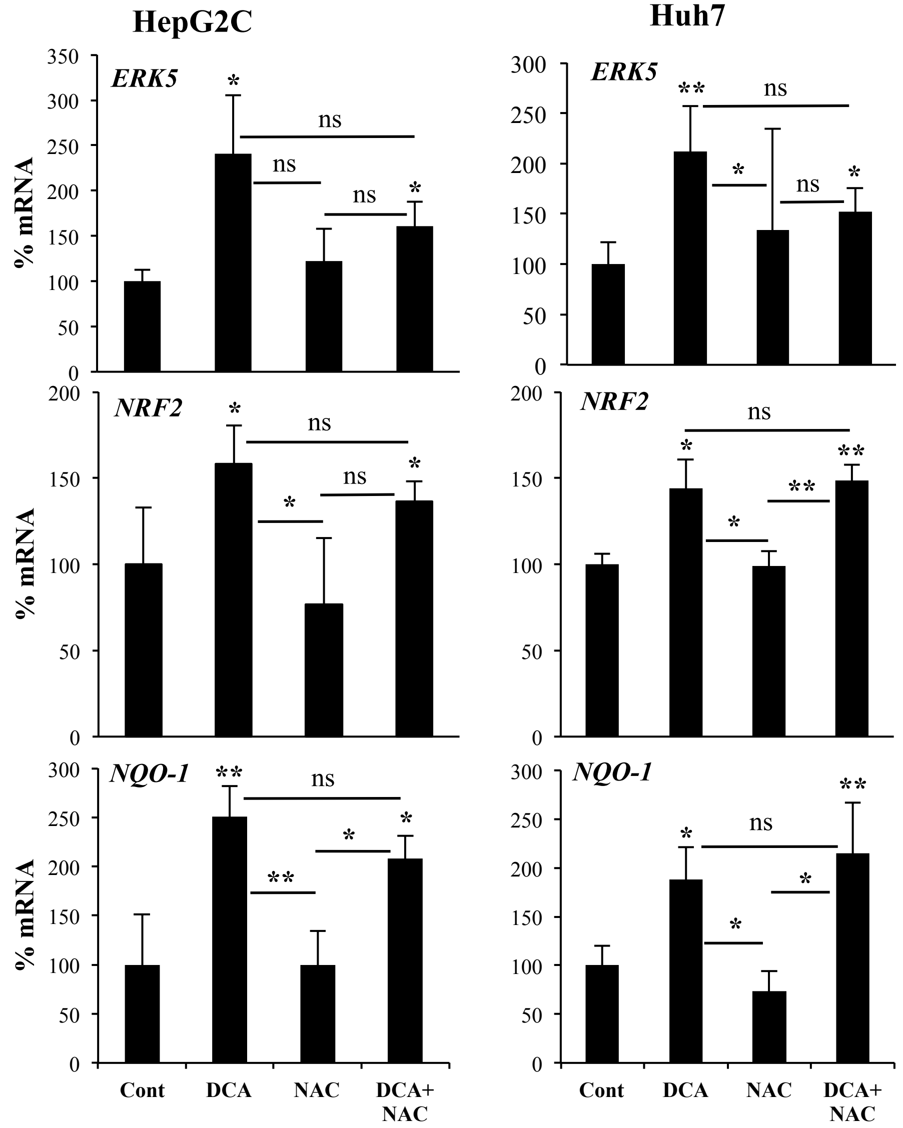

Supplemental Fig. 3. A) Jurkat and HepG2 cell lines were treated with 2 mM NAC 1 h before adding DCA (10 mM) for 24 h. mRNA was analyzed as described in Fig. 1. Results represent the means ± SD of 3 independent experiments performed in triplicate; statistics were performed using using One-way ANOVA with post-hoc Tukey test; * $p<0.05$, ** $p<0.01$, *** $p<0.005$. Treatments were compared to empty vector transfected cells (control) if not specified in the graph.

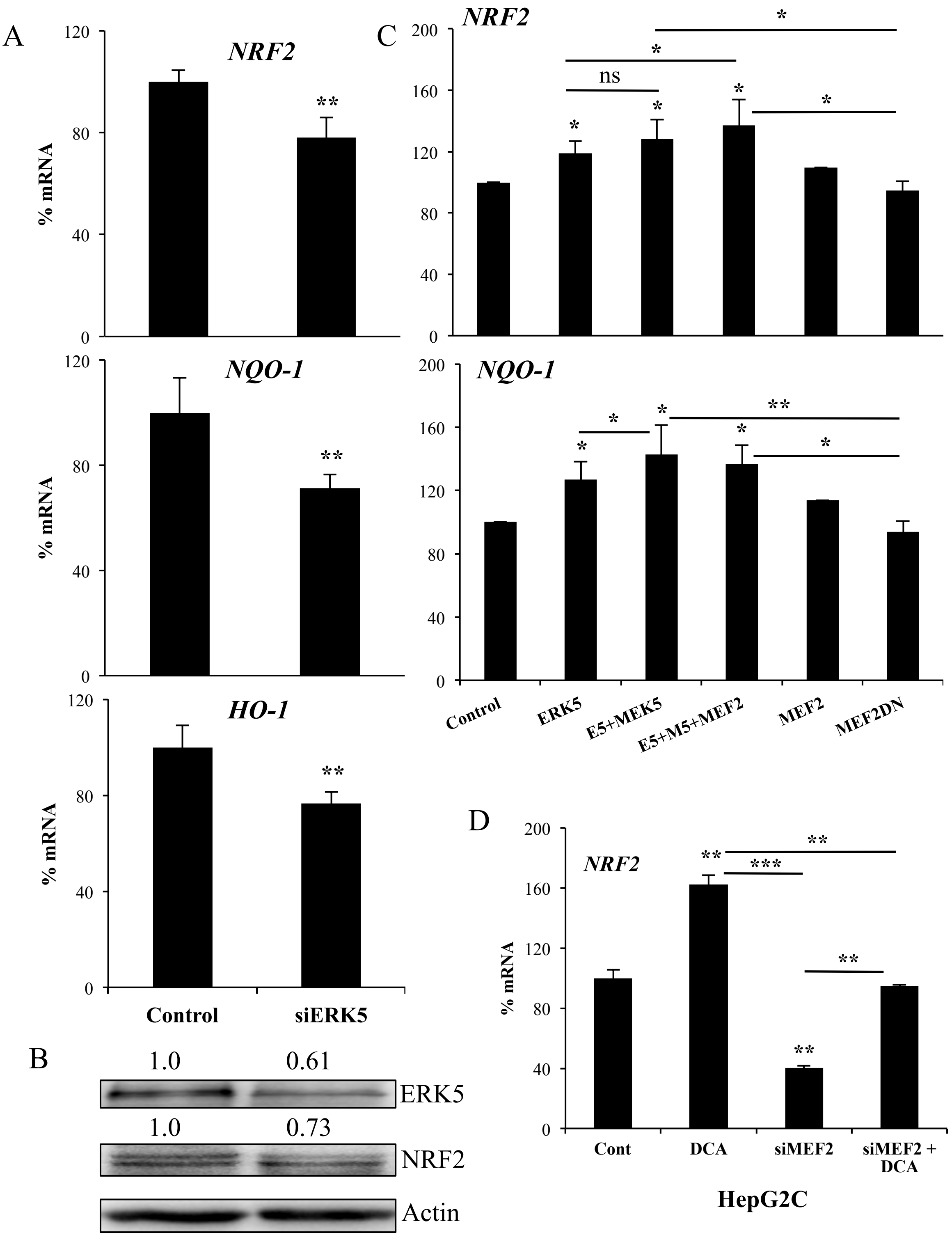

Supplemental Fig. 4. A) Huh7 cells were transfected with control siRNA or siRNA for ERK5. 72 h later *NRF2*, *NQO-1* and *HO-1* mRNA were analyzed. B) HepG2C cells were similarly transfected and ERK5 and NRF2 protein were analyzed. Numbers represent the ratio ERK5/actin. C) 10^7 Jurkat-TAg cells were co-transfected with 5 µg of the following vectors ERK5 wild type, a constitutively active MEK5 mutant (MEK5D), MEF2C and MEF2C with dominant negative function (MEF2-DN). 72 h after *NRF2* and *NQO-1* mRNA were analyzed. D) HepG2C cells were transfected with siMEF2 and 24h later were treated with DCA 72h later *NRF2* mRNA were analyzed. Data represent means ± SD; * p<0.05, ** p<0.01, *** p<0.005 student t-test compare to empty vector transfected cells (control).

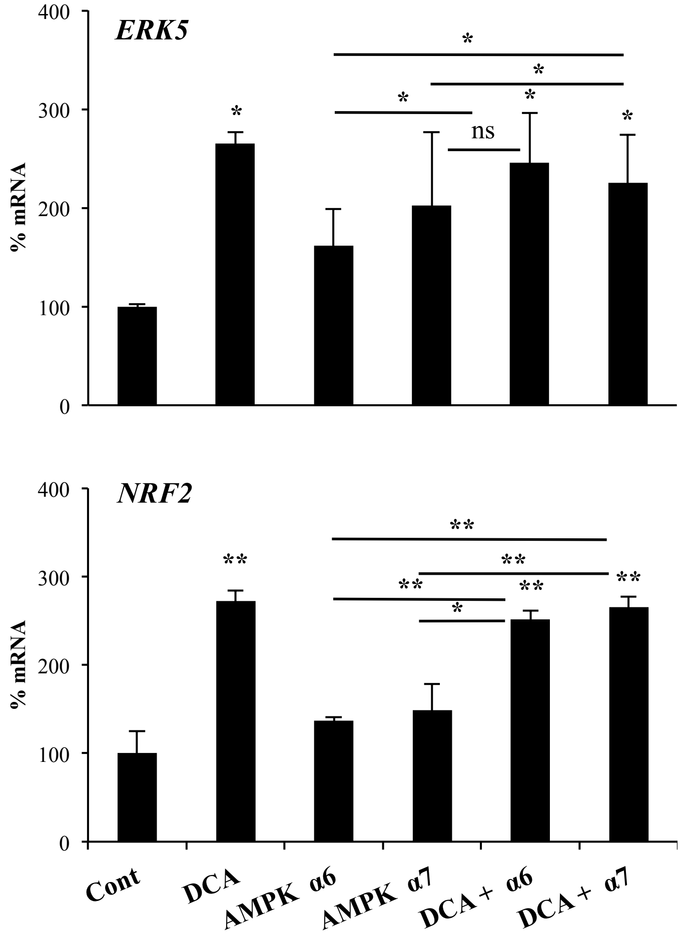

Supplemental Fig. 5. HCT116 cells were transfected with 2 small interference RNA (siRNA) for AMPKα or with control siRNA and treated with 20 mM DCA for 6 h before mRNA analysis. Data represent means ± SD; statistics were performed using using One-way ANOVA with post-hoc Tukey test; * $p<0.05$, ** $p<0.01$, *** $p<0.005$. Treatments were compared to empty vector transfected cells (control) if not specified in the graph.

Sup. Fig 5

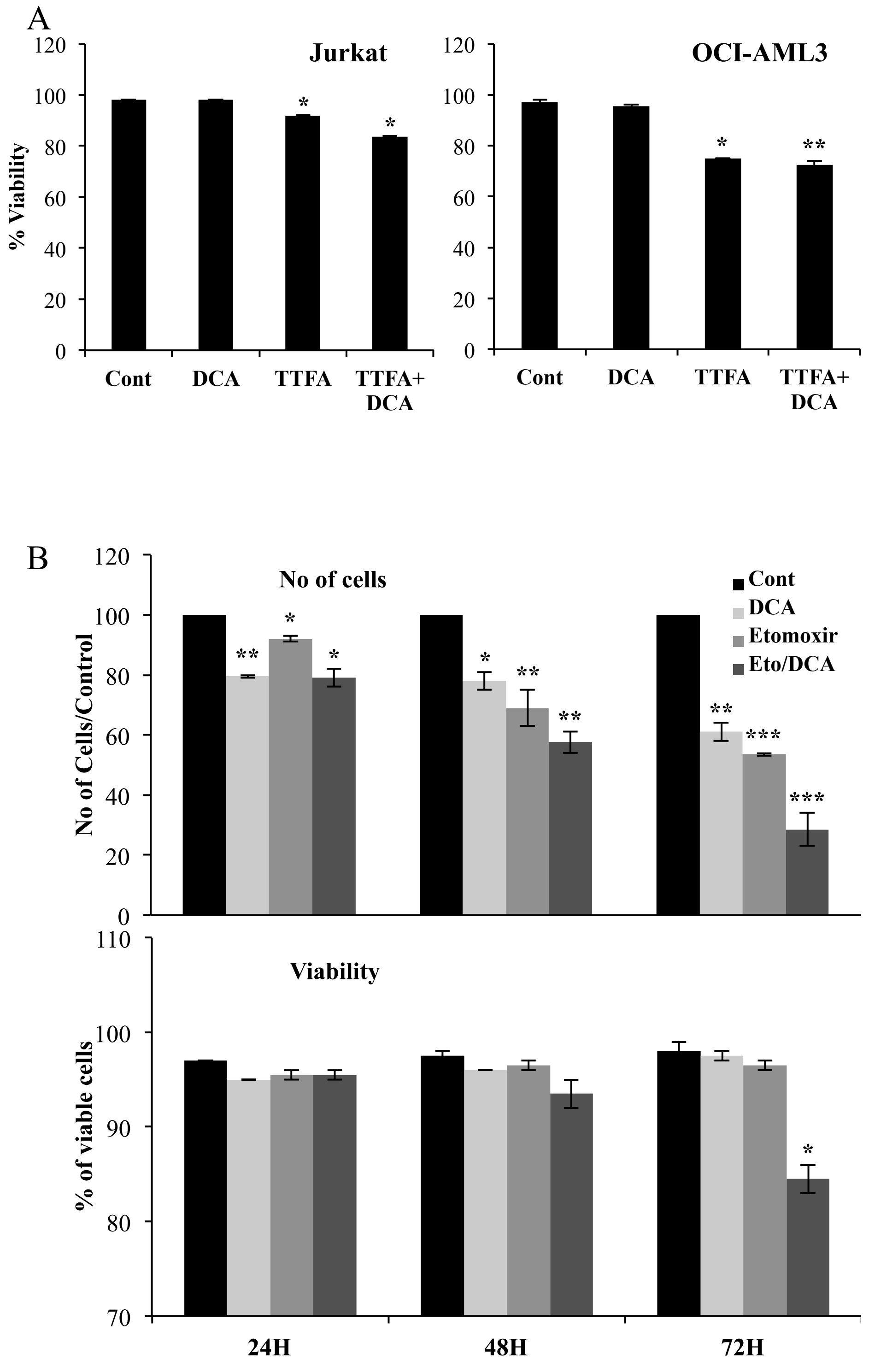

Supplemental Fig. 6. A) Jurkat and OCI-AML3 cells were treated with 10 mM DCA and/or 300 µM TTFA for 24 h and viability was quantified. B) OCI-AML3 cells were treated with 5 mM DCA and/or 100 µM etomoxir for the indicated times and cell number (upper) and viability (lower) were calculated.
